# Wearable Sensors and Motion Analysis for Neurological Patient Support

**DOI:** 10.3390/bios14120628

**Published:** 2024-12-19

**Authors:** Peter Dabnichki, Toh Yen Pang

**Affiliations:** 1 Mechanical, Manufacturing and Mechatronic Engineering, School of Engineering, STEM College, RMIT University, Melbourne, VIC 3000, Australia; 2 Biomedical Engineering, School of Engineering, STEM College, RMIT University, Melbourne, VIC 3000, Australia; tohyen.pang@rmit.edu.au

**Keywords:** wearable sensors, neurology patients, monitoring, rehabilitation, intelligent textiles

## Abstract

This work discusses the state of the art and challenges in using wearable sensors for the monitoring of neurological patients. The authors share their experience from their participation in numerous projects, ranging from drug trials to rehabilitation intervention assessment, and identify the obstacles in the way of the integrated adoption of wearable sensors in clinical and rehabilitation practices for neurological patients. Several highly promising developments are outlined and analyzed. It is considered that intelligent textiles are an attractive option, as they offer an esthetic outlook to and positive interaction with their users.

## 1. Introduction

Wearable technology has emerged as a valuable tool in the support of patients with long-term neurological conditions such as stroke, Parkinson’s disease (PD), multiple sclerosis, and other motor disorders [1,2,3,4,5,6].

For instance, rhythmic haptic cueing has shown promise in improving gait patterns for individuals with stroke, brain injury, and other neurological disorders [7].

Flexible bioelectronics, biosensors, and intelligent biosensing systems are being increasingly integrated into wearables, offering opportunities for continuous health monitoring, early diagnosis, personalized treatment, and improved lifestyle management for neurological patients [8,9,10].

Recent advancements in wearable technology have shown their critical role in the monitoring and management of neurological disorders, including Parkinson’s disease (PD), which is characterized by both motor and non-motor manifestations. These sensors are proving essential for early diagnosis, differential diagnosis, and the objective quantification of symptoms over time. The aim of this manuscript is to critically discuss the implications of implementing wearable technology in monitoring the health of individuals with neuropathy, focusing on recent advances in wearable sensors that enable more targeted rehabilitation and individualized care. We also provide recommendations for future developments in wearable sensors, informed by clinical studies with PD patients and the current literature, to enhance their usability in everyday life.

## 2. History of Wearables

**Figure 1:**
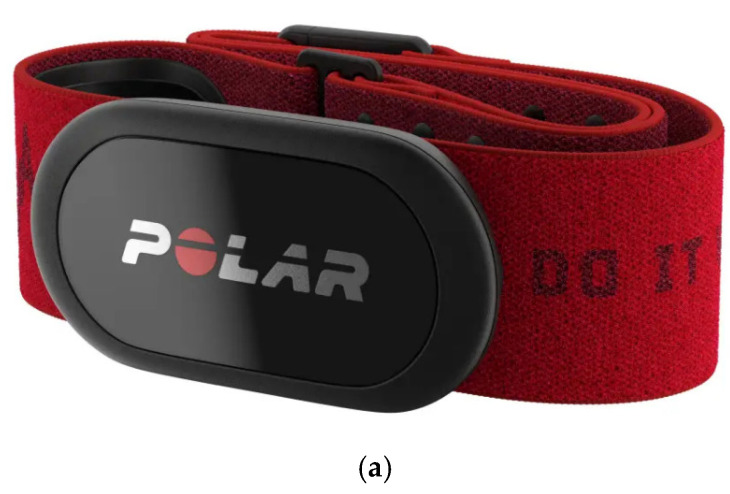

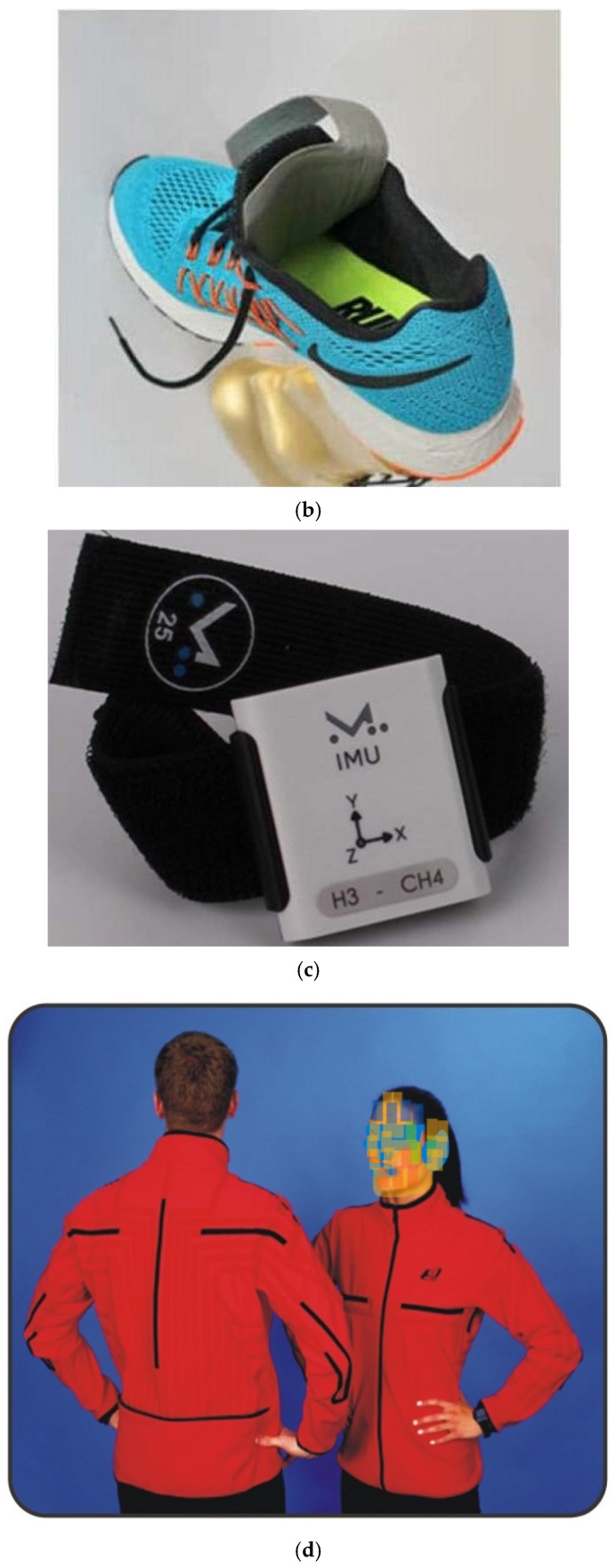
Wearable sensors: (**a**) Polar^®^ heart rate monitor; (**b**) Pedar^®^ pressure measurement insole; (**c**) Typical inertia measurement unit; (**d**) motion detection jacket by Philips and Levi Strauss.

The work on wearable clothing dates to the early 1990s, starting with the now legendary Polar heart rate monitor, through to highly sophisticated in-shoe pressure measurement systems such as Pedar^®^, and culminating in Philips’ and Levi Strauss’ launch of their groundbreaking motion detection jacket (see Figure 1). Although the idea at the time was to influence the emotions of the user while consuming entertainment, it had exciting potential for a user-friendly way of conducting motion analysis and potentially rehabilitation practices. An initial test by the authors showed the system’s potential for revolutionizing posture control and motion analysis tests. The great advantage of this type of wearable sensor battery was the convenience for the user and its fashion appeal that motivated participants and saved time by effectively installing the sensors instantaneously. The main drawback shown was the lack of cross-subject correlation as the mutual positioning of the body and sensors varied greatly. This shortcoming meant that the system could not be reliably introduced to rehab patients. The drawbacks of the sensors not being firmly placed directly on the body outweighed the benefits of not encumbering patients with many reflective sensors. In unpublished results, we further tried to enhance the jacket’s motion analysis features by adding reflective patches to conduct simultaneous sensor tracking. Despite the efforts, the noise level of the data proved an insurmountable obstacle, as the motion analysis models could not be reliably devised. One of the key issues is that in a non-controlled environment, execution protocols are not strictly adhered to, leading to a compromised data consistency that results in reduced intra-correlation. The overall outcome is that the diagnostic routines are still required to be performed in traditional clinical environments, reducing the appeal of wearables which become an extra chore for the patients.

**Figure 2:**
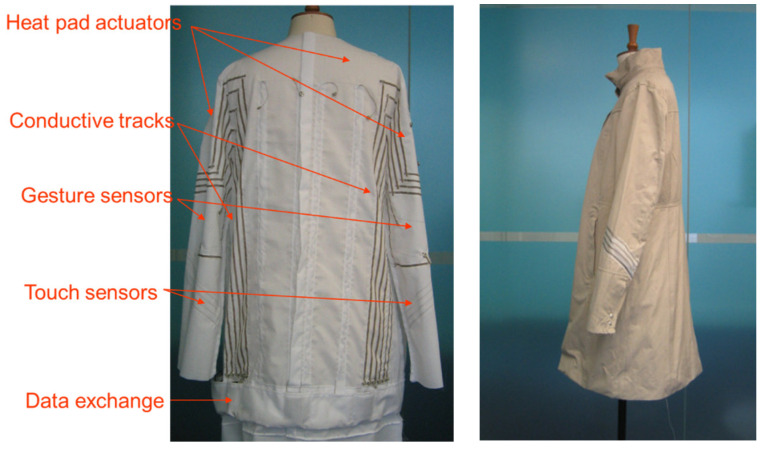
First-generation intelligent overcoat that contains sensors and actuators developed by the University of Arts London, UK.

Still, efforts did not go astray, as the potential of the sensors for emotionally responsive and comfort-providing clothing was pursued, and in the second half of the first decade of this century, the University of Arts (previously St Martins College of Arts) launched the first-generation intelligent textile product—a fashionable overcoat containing not only sensors but also actuators, providing positive responses reflecting the psychological and physiological status of the person wearing it (Figure 2). One such response is to play calming music when an agitated state is detected. Hence, the sensors are complemented by actuators that act upon a selected status, which requires some form of decision making; the most appropriate option was pervasive computing with a built-in processing power. Consequently, wearables have become inextricably connected to pervasive computing, the latter of which is gradually adopting AI techniques. In relation to clothing garments and apparel, advanced wearables are associated with intelligent textiles.

A further lesson we learned from these developments is that the issue of early adopters and reluctant users is of equal importance for wearables as for the introduction of new mass-use technologies. Unfortunately, a substantial portion of patients, especially neurological ones, fall into the latter category, and they would utilize wearables only as a last resort. However, when wearables are integrated into attractive fashion garments, their adoption is much smoother, and researchers enjoy working with highly active and willing participants. This helps to overcome a perceived stigma that affects patients with permanent conditions, which leads to depressive episodes and a reduced effectiveness of the proposed interventions.

**Figure 3:**
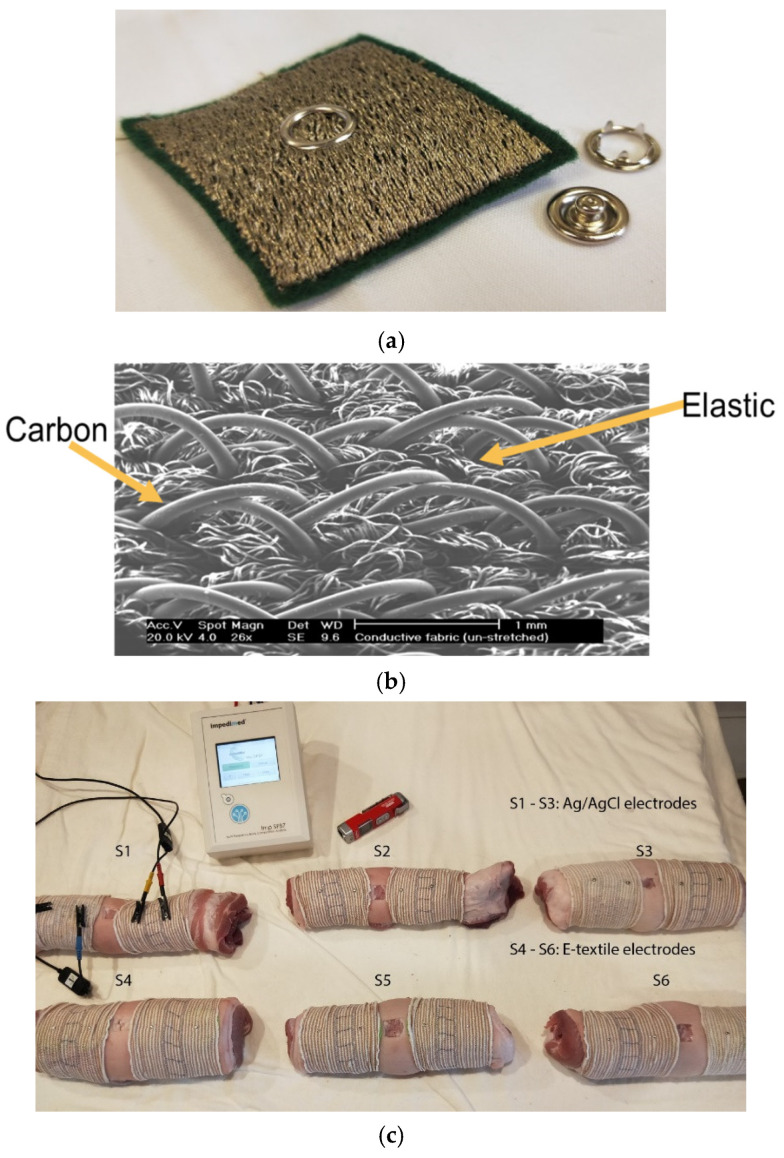
(**a**) Typical knitted sensor made of silver filaments typically attached to a garment; (**b**) more sophisticated textile sensors with carbon filaments; (**c**) comparative test between two sets of sensors on tissue conductivity.

When utilizing wearables in direct contact with the human body, there is a natural response that may be mild, such as the release of sweat or allergic reactions. On the emotional side, patients may feel tagged and even intimidated by the constant monitoring. Still, the first issue encountered is the affected thermal comfort, especially in warmer weather. In general, prolonged exposure to an irritant leads to an adverse physiological response [11]. This effect is exacerbated in neurological patients as their general neuro-physiological regulations are compromised. For example, Multiple Sclerosis (MS) sufferers exhibit random bouts of elevated temperature in various parts of the body. Hence, to avoid adverse thermal comfort effects, it is strongly recommended to have breathable patches attached to the body. However, the electric functionalities required by the sensors are usually in contradiction with this requirement, which is well known for the standard ECG sensors. The obvious way to circumvent this problem is to utilize textile sensors, as illustrated in Figure 3.

The conducting fibers are normally metal filaments [12], and at best, as illustrated in Figure 3, are carbon; this makes the textile inferior to the standard natural fibers we are accustomed to in our daily clothing garments, which is in addition to the usually adhesive contact of the sensors to the skin. The issue is still a challenge to the developers, and the usual strategy is to rely on an attenuated reaction threshold when the neural response is self-suppressed. This unfortunately does not necessarily occur in neurological patients, who are sometimes, like in the study that we reported recently, in an agitated state due to their treatment side effects.

Neurological conditions adversely affect posture, balance, mobility, flexibility, and coordination. All these attributes are measurable using traditional motion analysis tools and require short but well-controlled tests [13,14]. Standard tests pose little challenge, in technical terms, to be performed using wearables. However, their integration in everyday life proves difficult as data cannot be interpreted without activity context and environment details. Further to this, reliable interpretations still require health professional involvement and pose more challenges to the limited resources for these patients. This area is ripe for the introduction of AI tools that would allow personalized interpretation and intervention design. The motion-detecting sensors, such as the Inertia Measurement Units (IMUs), are most troublesome. They are sensitive to location variability, high frequencies caused by general interaction, and gravity acceleration.

## 3. Wearable Sensors and Patient Monitoring

### 3.1. Continuous Data Collection

The recent literature highlights that wearable sensors, such as accelerometers and gyroscopes, can effectively monitor Parkinson’s disease (PD), including tremors, bradykinesia, dyskinesia, and rigidity, providing continuous and objective data that are not achievable through standard clinical visits [3,15,16]. Wearable sensors can provide a real-time, continuous monitoring of neurological disorders, offering potential for continuous data collection in home and community settings [3,17]. These sensors are effective in capturing a range of physiological and movement-related variables in neurological conditions, such as epilepsy, PD, and stroke, enabling real-time assessments of motor symptoms, physical activity levels, and even seizure events [17,18]. For MS patients, wearables track fatigue, falls, and sleep quality, offering insights into daily challenges [19].

Advances in battery technology, movement sensors, and information technology have significantly boosted the field of objective measurement of movement using wearable sensors, allowing for continuous long-term monitoring [20,21]. One significant advantage of wearable sensors is their ability to provide accurate, low-cost, and non-invasive measurements, which contributes to the diagnosis, treatment, and long-term management of neurological disorders [5,20,22,23]. Additionally, wearable sensors can facilitate the monitoring of rehabilitation interventions and medication efficacy by tracking changes in motor function over time.

Despite the potential advantages, challenges remain, including the need for standardization, validation, and the regulatory approval of wearable sensor technologies [5,23,24]. For example, very few wearable ECG systems have been approved for use in clinical drug testing trials and no wearable biomechanical assessment systems are approved for clinical trials. Furthermore, there are no cross-validation requirements to allow for the transfer of raw data between systems. Hence, only processed data are compared. Furthermore, there are no vital sign wearable systems that are approved for clinical use. Hence, the gap between research and clinical practice is still existent.

### 3.2. Objective Measurements for Personalized Care

Wearable devices offer the potential to provide objective measurements that can reduce dependence on subjective reports, thereby improving the accuracy and reliability of clinical assessments and patient management. These devices can measure various motor outcomes such as gait, gross motor movements, and fine motor movements, offering a high degree of sensitivity and specificity in distinguishing between diseased individuals and healthy controls [15,25,26]. Data from wearables allow healthcare professionals to tailor treatment plans, adjusting medications and therapies based on real-time symptom tracking. For example, wearables have shown promise in quantifying motor symptoms and medication-evoked adverse symptoms in PD, as well as in assessing upper extremity activity and walking in stroke patients [3,27].

Wearables are generally well-accepted by patients, but there are concerns about the integration of these devices into daily life and the need for individualization to meet specific patient needs [3,28]. Further research is needed to establish their clinical utility, standardize measurement protocols, and address integration challenges to fully realize their potential in clinical practice [3,15,28]. Our experience with neurological patients is that they feel overwhelmed and controlled in every step of their life, leading to rejection. It is therefore essential to put the patient in control and integrate them in the decision making. An appropriate mode for this interaction is still actively sought.

### 3.3. Enhance Patient Engagment and Compliance

Wearables empower patients by providing them with tools to monitor their health, fostering a sense of control and encouraging adherence to treatment regimens [19]. Continuous monitoring enables the early detection of subtle changes in the patient’s condition, potentially allowing for timelier and more effective interventions. Still, the issue of the involvement of a neurology expert in such a continuous process without placing further burden on these specialists is largely unresolved, as it will require some deep change in the established clinical practices.

## 4. Impact on Patient Conditions and Well-Being

### 4.1. Impact on Medication

Wearables can help to monitor medication adherence and clinical features in patients with neurological conditions, but their clinical utility for decision making is not yet fully established.

Previous studies have demonstrated that wearables can effectively monitor medication adherence by quantifying movement patterns and symptoms in patients with neurological conditions like Parkinson’s disease. This allows for remote monitoring and early warnings regarding patient safety [3,29]. Wearables are used to quantify medication-evoked adverse symptoms in both laboratory and free-living environments. This helps in understanding the impact of medication on daily activities and overall health [3].

Patients find, in general, that wearables are acceptable for integration into daily life. However, there is a lack of confidence in the technology, and there is a need for individualization to cater to specific patient needs and preferences [3].

### 4.2. Impact on Rehabilitation Interventions

Wearable technologies are increasingly recognized for their potential to improve mobility and functional independence for patients with chronic neurological conditions. By facilitating the continuous monitoring of health metrics, such as step count, gait symmetry, and upper extremity function, these devices provide valuable insights into patient progress [4,30]. Moreover, the capability for real-time biofeedback, as demonstrated by devices with auditory or visual feedback, can reinforce positive movement patterns, promoting better motor learning and engagement in rehabilitation activities, which has been shown to improve balance and gait in patients with neurological disorders [31]. Devices that offer haptic feedback have also demonstrated immediate improvements in gait parameters, such as stride length and walking speed, which are essential for enhancing mobility and independence [32]. While wearables show promise in improving rehabilitation outcomes, challenges remain in ensuring their usability and effectiveness in diverse real-life contexts. Further research is needed to optimize these technologies for broader application in chronic neurological rehabilitation.

### 4.3. Patients’ Self-Esteem and Quality of Live

Wearable technologies show promise for improving self-management and potentially self-esteem in patients with chronic neurological conditions. Wearables can measure symptoms such as tremors, gait, and balance, helping patients and clinicians track disease progression [33]. Some devices provide cues or stimulation to improve movement, potentially increasing patients’ confidence in their mobility [34].

While the potential for wearables to improve self-esteem in patients with chronic neurological conditions is promising, more research is needed to fully understand their impact. Studies show mixed results, with some patients experiencing increased confidence and independence, while others may feel overwhelmed or overly reliant on technology [34,35]. As these technologies continue to evolve, it will be crucial to assess their long-term effects on patients’ psychological well-being alongside their physical health benefits.

### 4.4. Assessment of Patient Conditions and Well-Being

The large volume of data that wearables produce when continuously used by patients require expert’s time and deep analysis. In the past, the authors tackled the problem of large volumes of the ECG data that are mandatory in drug trials. Most of the computational advances are in a similar category when incidents need to be detected in large volumes of data, and they are normally exhibited by a sharp change in the signal pattern. This means that a large proportion of the data is discarded, and only specific episodes kept and analyzed. However, in the case of neurology, sharp changes in patients does not occur and one needs to detect miniscule changes over long-term trends; this is a harder obstacle, as such expertise is only reached by a few top-tier experts. Hence, devising AI-type tools would require high integration between such experts in long-term projects, a task with prohibitive cost implications.

## 5. Challenges, Knowledge Gaps, and Considerations

Current wearable devices face challenges in achieving user-friendly designs, ensuring data security and privacy, and meeting industry standards [36,37,38].

### 5.1. Privacy, Security, and Standardization

Dealing with challenges regarding data privacy and management [39] and ensuring the privacy and security of patient data are paramount, especially with the continuous monitoring of patients.

### 5.2. Long-Term Engagement

Wearable sensors often face challenges in adherence, especially among elderly patients who may experience technology fatigue or usability issues [18].

### 5.3. Knowledge Gaps

**Figure 4:**
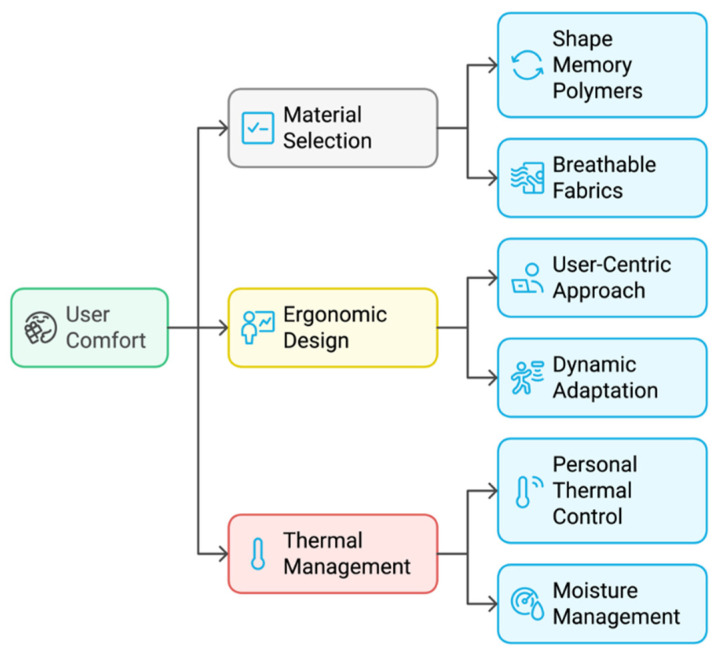
Key aspects that contribute to the enhancement of user comfort in wearable sensor design.

**Figure 5:**
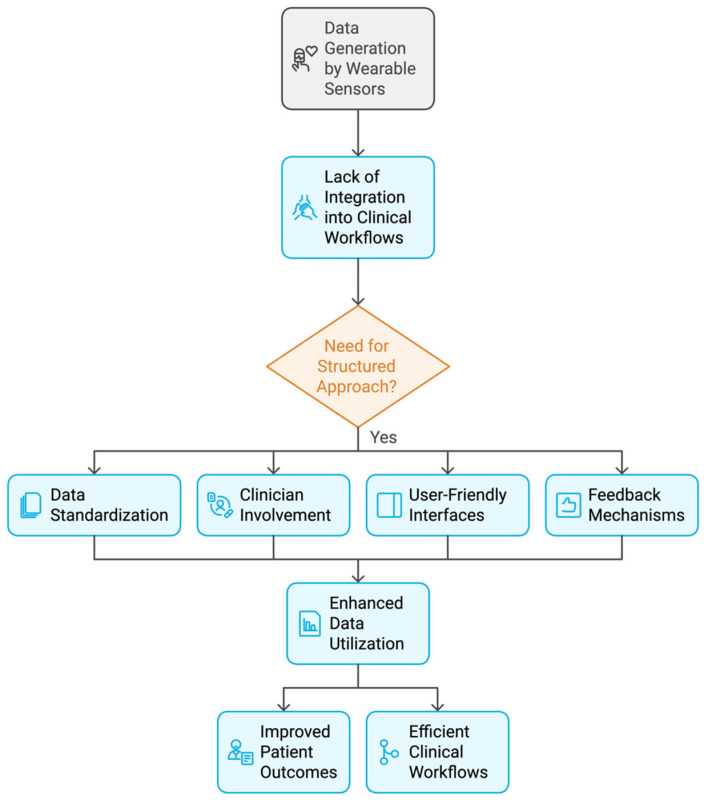
The challenges and opportunities for the integration of sensor-generated data into clinical workflows for improved patient outcomes and clinical workflows.

Drawing from our current work involving wearable gait analysis and rehabilitation studies with PD patients [14,40], and insights from the current literature, we identified the following areas in need for further improvement:

− User comfort: current sensors are built for functionality with little attention to users’ response to texture and tactility. User experience and the acceptance of wearable sensors are influenced by material selection and textile properties [41,42], ergonomic design, and thermal management (Figure 4).
− Wearable sensors and sustainability: there are still issues with storage and, most importantly, hygiene requirements, especially for devices in continuous skin contact. Although some sensors retain usability after a single washing, they quickly lose sensitivity after a few cycles. The emphasize on sustainability and eco-friendly wearable technology solutions need to be addressed.
− Clinical context integration: sensor data are not essential unless placed in a clinical context. Current medical practices cannot accommodate the sizeable data volumes, and they remain, at best, under-utilized. Despite the potential benefits of wearable sensor data, the integration of sensor-generated data into clinical workflows presents both challenges and opportunities. Figure 5 illustrates a suggested framework to address these challenges. We should consider establishing standardized formats for sensor data, which are essential to ensure consistency and facilitate seamless interpretation across various devices and platforms. Regular interactions between patients and healthcare professionals are critical for contextualizing these data, thereby enabling informed decision making and the formulation of personalized care plans.
− Patient engagement and psychological impact: the development of wearables is not a technical challenge anymore. The current challenge is how to collect, interpret, and utilize the data without placing a burden on medics and causing adverse psychological effects on patients. Current research reporting practices places its focus on statistical interpretation and methodology. For example, we have come across a paradox, where patients showed improvement but their satisfaction and self-esteem were lower than the group that had an inferior outcome. This simply means that our technologies would be discarded as soon as the trial is over. It is also evident that spreading effective practices across the patient population is difficult, as the very nature of clinical trials requires the recruitment of highly motivated individuals who do not reflect the general population’s attitude.

## 6. Recommendations for Everyday Use of Wearable Sensors

Advances in flexible electronics, biosensors, and AI algorithms are driving the development of more sophisticated and personalized wearable devices [41,42]. Still, there are issues with the individual responses of acceptance and adherence that prevent the proliferation of such devices in clinical practices. The global issue of adoption by patients should not disguise shortcomings in the current devices that suppress general implementation in rehab practices.

1. Irritation to skin when worn for a prolonged period is an issue that should be at the forefront of development, i.e., when the sensor is conceived.
2. Hygiene maintenance is still a major issue, and most wearables lose operability after only a few wash cycles, if they allow washing at all.
3. Esthetics is quite important, as patients prefer that devices do not have a “medical look” so that they are not perceived as sick by the society. Art design experts have a much better ability to attract users and should be approached when needed.
4. Communicative protocols and data storage. Patients are wary of cloud storage and do not like feeling like they are not the owner of their own data. Hence, local storage options should be considered when possible.
5. Textile and other fashion apparel is more attractive than usual devices and more likely to promote compliance.
6. Metal fibers in textile sensors tend to cause adverse reactions, even when coated with nylon and other similar materials.

Finally, wearables are preferably used for targeted data collection and so should be limited in numbers. The old idea of a body area network containing a high number of sensors is unwanted by users and impractical for researchers. This, in turn, means that wearable sensor data need to be put into context by highly sophisticated software, while sensors should be carefully clustered to allow context reconstruction. We personally believe, based on our experience, that adoption chances are greatly enhanced by the provision of actuators that, for example, provide heating or cooling. They provide tangible benefits that patients cherish but also provide a challenge for developers who need to address the issue of energy harvesting. Still, with these issues in mind, we feel that intelligent textiles integrating clusters of wearable sensors are the most promising avenues for research.

## Data Availability

No new data were created.
